# Pannexin Channel Regulation of Cell Migration: Focus on Immune Cells

**DOI:** 10.3389/fimmu.2021.750480

**Published:** 2021-12-16

**Authors:** Paloma A. Harcha, Tamara López-López, Adrián G. Palacios, Pablo J. Sáez

**Affiliations:** ^1^ Centro Interdisciplinario de Neurociencia de Valparaíso, Instituto de Neurociencia, Facultad de Ciencias, Universidad de Valparaíso, Valparaíso, Chile; ^2^ Cell Communication and Migration Laboratory, Institute of Biochemistry and Molecular Cell Biology, Center for Experimental Medicine, University Medical Center Hamburg-Eppendorf, Hamburg, Germany

**Keywords:** cell communication, leukocytes, cancer, inflammation, Ca^2+^ signaling, amoeboid migration, mesenchymal migration, mechanotransduction

## Abstract

The role of Pannexin (PANX) channels during collective and single cell migration is increasingly recognized. Amongst many functions that are relevant to cell migration, here we focus on the role of PANX-mediated adenine nucleotide release and associated autocrine and paracrine signaling. We also summarize the contribution of PANXs with the cytoskeleton, which is also key regulator of cell migration. PANXs, as mechanosensitive ATP releasing channels, provide a unique link between cell migration and purinergic communication. The functional association with several purinergic receptors, together with a plethora of signals that modulate their opening, allows PANX channels to integrate physical and chemical cues during inflammation. Ubiquitously expressed in almost all immune cells, PANX1 opening has been reported in different immunological contexts. Immune activation is the epitome coordination between cell communication and migration, as leukocytes (i.e., T cells, dendritic cells) exchange information while migrating towards the injury site. In the current review, we summarized the contribution of PANX channels during immune cell migration and recruitment; although we also compile the available evidence for non-immune cells (including fibroblasts, keratinocytes, astrocytes, and cancer cells). Finally, we discuss the current evidence of PANX1 and PANX3 channels as a both positive and/or negative regulator in different inflammatory conditions, proposing a general mechanism of these channels contribution during cell migration.

## 1 Introduction

Cell communication and cell migration are key phenomena for development, tissue repair, and immune response; thus coordination of these responses are key for sustaining life (1–4). Indeed, a fine coordination of leukocyte communication is required for migration to clear an infection, or recruit other migrating cells towards an injury site. Interestingly, immune cells use different migratory strategies associated with their immune function and location (5–7). For example, under resting conditions immune cells would randomly patrol the tissue, but upon activation undergo directional migration to reach the secondary lymphoid organs (8). Despite presenting unique features, immune cell migration follows the general rules of cell migration, and depends on cytoskeletal dynamics (actin, non-muscular myosin II [MyoII]), and microtubules, as described in detail in the following reviews (6, 9–11). The study of immune cell migration is directly linked to development of new techniques to monitor the behavior of these cells in their native microenvironment (5, 12, 13), although this is still very challenging. However, researchers have developed *ex vivo* (i.e., tissue slices), and *in vitro* systems that mimic some tissue properties (i.e., confinement, properties of the extracellular matrix, etc). Thus, motility has been studied in models with different levels of microenvironment complexity (i.e., 1D, 2D, and 3D), topographies (that do or do not impose cellular deformation), or that mimic their transmigration through tissue layers (6, 13, 14).

### 1.1 Danger Signals and the Role of Purinergic Signaling

Immune cell migration is also controlled by microenvironmental chemical cues, such as chemokines and danger signals, affecting cell positioning along the tissue (15–17). Danger signals are molecules that trigger the immune system, and are classified due to their origin: damage-associated molecular patterns (DAMPs) [such as extracellular adenosine-5′-triphosphate (ATP)], and pathogen-associated molecular patterns (PAMPs) [such as lipopolysaccharide (LPS) (16). Both DAMPs and PAMPs trigger immune cell maturation, and expression of receptors (i.e. chemokine receptors) promoting directional migration towards the injury site. Interestingly, extracellular ATP acts both as a DAMP when released from damaged cells, or as signaling molecule when released from healthy cells. In both cases, ATP activates purinergic receptors (P2) triggering subsequent downstream signaling that depends on the activated receptor.

P2 receptors are classified into ionotropic (P2X) receptors that allow calcium ions (Ca^2+^) influx, and metabotropic (P2Y) receptors that trigger Ca^2+^ release from intracellular stores (18). Both families of P2 are widely expressed on immune cells, controlling a plethora of functions (18), including cell communication. The activation of specific P2X and P2Y receptors family members depends on the exposure time and agonist concentration, which allows the spatiotemporal regulation of the signaling (19). In addition to the differential activation of P2 receptors the concentration of adenine nucleotides, such as ATP, is integrated by immune cells and decoded as low or high inflammatory state (18). Immune cells use different cell communication mechanisms dependent or independent on cell contacts, which amplify signals according to that inflammatory state (2, 6, 7, 20). The release of small molecules *via* plasma membrane channels, such as connexins (CXs) and pannexins (PANXs), and its coupling to purinergic signaling represents a widely used mechanism for cell communication, which plays a role in paracrine (between cells) and autocrine (cell autonomous) signaling (2, 18, 21).

### 1.2 Cell Polarity and PANX-Dependent Signaling

CXs and PANXs are membrane proteins that allow the exchange of small molecules (i.e., glucose, ATP) and ions between the cytoplasm and the extracellular milieu (22). Upon docking, CX channels of adjacent cells form intercellular channels that connect their cytoplasm, namely gap junction channels, although PANX channels until now are shown to form only hemichannels at the plasma membrane. The latter puts PANX channels and purinergic signaling in the center stage of both cell-autonomous signaling and contact-independent cell communication (23), which are required for efficient motility. Migrating cells use cellular polarity, the asymmetric organization of intracellular components, as the navigation system that determines the direction of migration (6, 10, 11). Cell polarity is dynamically set by changing the position of organelles, cytoskeleton, and signaling proteins (10, 24). Thus, polarization of the actin cytoskeleton allows the establishment of a front-rear migration axis, which subsequently polarizes other proteins (10). Interestingly, F-actin and its regulator Arp3 directly interact with PANX1 (25–27), suggesting that F-actin polarization and nucleation of new microfilaments might directly control PANX1 localization. Indeed, actin flow and polarization permits the concomitant polarization of PANX1 to the leading edge in migrating immune cells (28–30). Similarly, PANX3 stability at the plasma membrane requires intact actin cytoskeleton (25), suggesting that a similar mechanism might take place during cell migration.

Altogether, the co-polarization of PANXs with the actin cytoskeleton, and their indirect functional impact on microtubules, *via* protein-protein interaction with a microtubule stabilizer (31), could imply a polarization of the PANX-dependent signaling. Therefore, ATP and other molecules that permeate through PANX-channels will be released in a polarized manner that might sustain the cell polarity and direction of migration (28, 30, 32).

In this review, we summarize the contribution of PANX1 channels during cell migration. The first part of this review is focused on different immune cells, as example of PANX1 channel contribution to leukocyte migration and recruitment. Then, we describe PANX1 and PANX3 contribution to migration of non-immune cells, such as astrocytes, skin cells, and cancer cells, as well as the few available evidence for the PANX2 and its putative role during cell migration. Afterwards, we dedicate a final section of PANX1 contribution to cell migration during neuroinflammatory conditions, and aging.

## 2 Immune Cell Migration

Most leukocytes use amoeboid migration to move within tissues. This migration mode is characterized by limited adhesion to the extracellular matrix with little (or non-) proteolytic activity, preventing extracellular matrix modification (5). Therefore, in order to undergo fast migration after damage, immune cells must deform their cellular body while facing microenvironment obstacles (6, 9). Leukocytes highly rely on acto-myosin cytoskeleton contractility, and mechanosensitive channels, including PANXs channels (6, 9, 30). Interestingly, PANX1 is required for homing of bone-marrow derived immune cell precursors (33), suggesting that these channels are required from early stages of development. Moreover, since leukocytes reside in different tissues and are exposed to different mechanical and chemical signals, these cells exhibit different migration strategies. Despite this cell-specific migratory behavior, the contribution of PANX1 channels to purinergic and Ca^2+^ signaling, and to cytoskeleton regulation is well established. In general PANX1 channels are positive regulators of immune cell migration as summarized in this section (**Table 1** **)**.

**Table 1:** Summary of CXs and PANX channels contribution to immune cell migration.

Cell type	Channel	Channel blockers//receptor inhibitors	P2R, AR	Migratory stimuli	Migration techniques	Ref.
**DCs**						
BMDCs (m)	CX43	αGA, CX43 KO	n.e.	CCL21	3D chemotaxis in collagen	(34)
DEC205^+^ DCs (m)	CX43, CX45	n.e.	n.e.	BaCl muscle damage	*In vivo* homing to LNs	(35)
BMDCs (m)	PANX1	PANX1 KO// A-740003, Apyrase, BAPTA, KN-62, oATP	P2X_7_	Extracellular ATP	microchannels, 3D collagen, *in vivo* homing to LNs	(32)
**Macrophage**						
Peritoneal macrophages (m)	PANX1-indep.	PANX1 KO//P2Y2 KO, P2Y12 KO, AR-C69931 MX, 8-SPT, MRS-2179, NF449	P2Y_2_, P2Y_12_	2D chemotaxis	2D chemotaxis	(36)
Cortical CX3CR1^+^ microglia (m)	und.	Cbx, FFA//Apyrase, RB2, PPADS, Suramin	P2Y	Laser ablation, extracellular ATP	*In vivo* recruitment to injury site 2PEF	(37)
Retinal CX3CR1^+^ microglia (m)	PANX1	Pbc//Apyrase, Suramin	P2	AMPA	*Ex vivo* retinal explants process dynamics	(38)
Cortical CD68^+^ microglia (m)	PANX1	Trovafloxacin	n.e.	Controlled cortical impact	*In vivo* recruitment to injury site	(39)
BV-2 microglia cell line	PANX1	Trovafloxacin, BBFCF, ^10^PANX1	P2	C5a	Transmigration in transwells	(39)
**Monocytes**						
PBMCs (m)	CX43	αGA, octanol	n.e.	MCP-1	Transmigration through endothelial layer	(40)
PBMCs (h), THP-1 cell line	PANX1?	P2Y_6_ siRNA, BMSCCR222, PTX, U73122, BAPTA, Apyrase, MRS2578	P2Y_6_	CCL2, fMLP	Transmigration in transwells	(41)
**Neutrophils**						
PMNs (m)	PANX1	Cbx, ^10^PANX1, P2Y2 KO, DIDS, Suramin	P2Y_2_	fMLP	Chemotaxis in 2D release from a pipette	(42)
PMNs (h), HL-60 cell line	PANX1	Cbx, ^10^PANX1//CSC, CGS21680, H89	A2a	fMLP	Chemotaxis in 2D release from a pipette	(43)
Lung neutrophils (m)	CX43	CX43+/-, Gap26	n.e.	LPS	Counting of *in vivo* homing to the lungs	(44)
HL-60 cell line	CX43 (as neg. reg.)	Gap19, ^10^PANX1//P2Y1 KO, SB 203580	P2Y_1_	LPS	Chemotaxis in 2D confined under agarose	(45)
Neutrophils (z.f.)	CX43	Cbx, CX43 morphans, lyz:cx43DN-T2A-mCherry	n.e.	Laser ablation	*In vivo* recruitment to injury site 2PEF	(46)
**T cells**						
Innate lymphoid cells (ILCs)	PANX1	PANX1 KO	n.e.	House dust mite	No direct effect	(47)
CD4^+^ T cells (m)	PANX1	PANX1 KO	n.e	House dust mite	*In vivo* recruitment to lung	(47)
CD4^+^ PMBCs (h)	PANX1	Cbx, PANX1 KD, ^10^PANX1//Apyrase, suramin, CCCP	P2X_4_	CXCL12	Chemotaxis in 2D, transmigration in transwells	(29, 48)
CD4^+^ splenocytes (m)	PANX1	PANX1 KO	n.e.	Tissue CXCL12	Counting of *in vivo* spinal cord recruitment	(29)
CD4^+^ T cells (m)	PANX1	Cbx	P2Y_10_	CCL19	Transmigration in transwells	(49)
CD3^+^ cells (m)	PANX1	Pbc	n.e.	und.	Counting of *in vivo* spinal cord recruitment	(50)
**Other**						
Brain mast cells (m)	CX43, PANX1	n.e.	n.e.	Amyloid β-Peptide	Cortical recruitment in APPswe/PS1dE9 Alzheimer’s model.	(51)
HSPCs (m)	PANX1	^10^PANX1	und.	G-CSF, AMD3100	*In vivo* homing to tissue	(33)

2PEF, 2-photon excitation microscopy; aGA, a-glycyrrhetinic acid; AMPA, a-amino-3-hydroxy-5-methyl-4-isoxazole propionic acid; BMDCs, Bone-marrow derived DCs; Cbx, Carbenoxolone; HSPCs, hematopoietic stem/progenitor cells; FFA, flufenamic acid; G-CSF, granulocyte colony-stimulating factor; h, human; fMLP, N-formyl-Met-Leu-Phe peptide; KD, knock-down; KO, knock-out; LPS, lipopolysaccharide; LN, lymph node; m, mouse; N.E., not evaluated; oATP, oxidized ATP; PBMCs, peripheral blood mononuclear cells; PTX, Pertussis toxin; PMNs, Polymorphonuclear cells; Pbc, Probenecid; und., undetermined; z.f., zebra fish.

### 2.1 Neutrophils

Neutrophils, key components of the innate immune system, are polymorphonuclear phagocytic cells found in the bloodstream. Upon danger signal (i.e. chemokine/cytokine detection after infection or injury) neutrophils leave the bloodstream to invade vascular tissues (52). In the tissue, neutrophils have phagocytic activity, producing reactive oxygen species, forming neutrophil extracellular traps (NETs), and releasing cytokines, chemokines and bactericidal peptides (17, 53). PANX1 channels are highly expressed in neutrophils and contribute to their activation (42, 43, 54, 55). For instance, after exposure to non-esterified fatty acids (i.e., oleic and linoleic acid), NET formation requires activation of P2X_1_ receptors by extracellular ATP, released *via* PANX1 channels (55).

**Figure 1:**
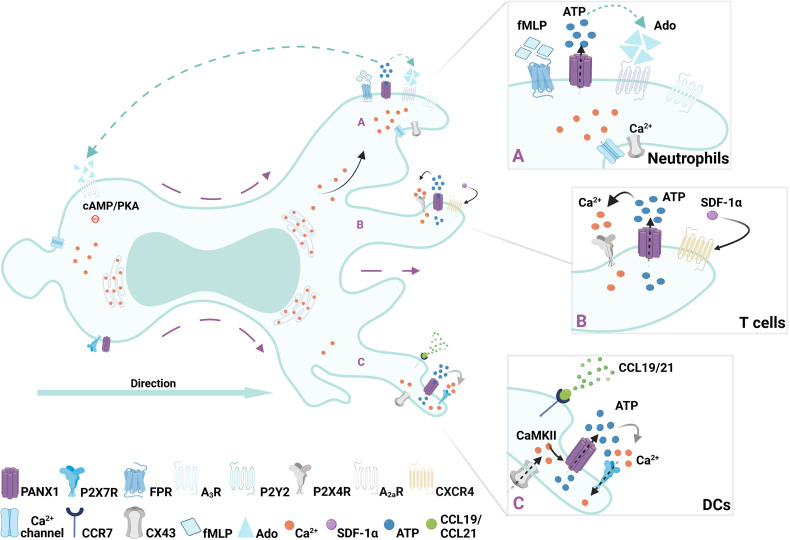
Regulation of immune cell migration by PANX1 channels. Redistribution of surface PANX1channels have been described during migration of immune cells. **(A)** In neutrophils after FMLP gradient sensing, opening of PANX1 channels polarize towards the leading edge (right side of the cell) allowing ATP release, subsequently activating local purinergic P2Y_2_ and -upon ATP degradation- adenosine A3 receptors. Then, at the rear of the migrating cell, activation of the adenosine A2a receptors by adenosine (Ado) promotes inhibitory cascades mediated by cAMP/PKA, leading to an orchestrated cytoskeleton rearrangement required for migration. **(B)** In T cells activation of CXCR4 receptor with SDF-1 triggers controlled burst of ATP after PANX1 channels opening, which is accompanied by mitochondria and P2X_4_ translocation to the leading edge where Ca^2+^ influx occurs. **(C)** In DCs, ATP-induced fast migration requires an autocrine feedback loop between PANX1 channels and P2X_7_ receptor, which triggers Ca^2+^ influx and subsequent activation of CaMKII, which maintains PANX1 channels opened.

Indeed, adenine nucleotide release and purinergic signaling cascade activation are key for neutrophil migration, particularly when the release of ATP is used as a navigational cue (43, 54). Accordingly, several purinergic receptors regulate neutrophil migration, including P2X1 (45), P2Y_2_ (42, 54, 56), P2Y_6_ (57), P2Y_11_ (58), P2Y_14_ (59), A1 (60), and A2a (61). Interestingly, components of the purinergic signaling that contribute to migratory response are polarized (**Figure 1**), and therefore provide a spatio-temporal dimension to this phenomenon (43, 54).

Neutrophils migration towards a N-formyl-Met-Leu-Phe (FMLP) gradient, which mimics a bacterial-induced chemotactic response, also depends on extracellular ATP sensing. During FMLP-induced migration ATP is released in a polarized manner, as PANX1 is polarized to the leading edge together with F-actin (42). This polarization towards the leading edge relies on the direct interaction between F-actin and the C-terminus of PANX1 previously described (25, 27). ATP released *via* PANX1 leads to the subsequent activation of P2Y_2_ and A3 receptors, which are also localized at the leading edge generating an autocrine feedback loop required to maintain the polarization of the cell and to amplify the gradient sensing (42, 54). Interestingly, PANX1 channels also contribute to the inhibitory signals at the rear of the cell (43). The continuous degradation of ATP by ectonucleotidases produces adenosine, which activates adenosine A2a receptors at the cell rear, leading to intracellular cAMP/PKA signaling, and inhibiting excitatory signals from the leading edge (43). Since adenosine activates both A3 and A2a, PANX1 indirectly contributes to excitatory and inhibitory signals required for cell migration, as shown in **Figure 1**. So far, polarization of positive and negative signals regulated by PANX1 has only been reported on neutrophils, although is tempting to assume it as a general mechanism for immune cell migration.

Moreover, neutrophils present additional pathways to release ATP, such as CXs and tweety family member 3 (TTYH3) maxi anion channels (2, 42), but it seems that different channels and purinergic receptors are recruited depending on the stimuli of the immune response. For example, in a mouse model CX43 hemichannels contribute to LPS-induced neutrophil recruitment in the lungs (44). Similarly, CX43 hemichannels provide a path for ATP release that promotes neutrophil swarming during laser wound tissue damage in zebrafish (46). However, when CX43-mediated ATP release is coupled to activation of P2X_1_ receptors, this reduces the migration of human neutrophils and HL-60 neutrophil-like in an under agarose assay, and then acts as a stop signal (45).

Overall, PANX1 contributes to neutrophil cell migration by establishing a polarity axis, which is supported by re-localization of the actin cytoskeleton, and the purinergic signaling-related proteins involved in the migratory response. However, the putative role of PANX2 and PANX3, and in pathological conditions remains largely unexplored.

### 2.2 T Cells

T cell migration is a key step during the adaptive immune response, and its pattern varies with the activation state and the microenvironmental context. Before antigen exposure, and during antigen-presenting cells (APC) search in the lymph nodes, T cells have a diffusive and random migration (Brownian type), whereas less diffusive chemotactic movements (Lévy type) is exhibited by recently activated T cell to migrate into secondary lymphoid organs for priming; or highly directional migration (ballistic) induced by haptotaxis cues and chemotaxis gradient caused by cognate APC at the peripheral tissues (8). Adaptive immune response progression requires purinergic signaling to modulate T cell functions (28). For example, P2X_7_ receptor modulates the balance between the number of differentiated Th_17_ and T_reg_ lymphocytes (62). Accordingly, PANX1 and purinergic receptors are essential for T cell activation and cell death (63–65). In both cases, ATP is released through PANX1 channels activating purinergic receptors, triggering intracellular cascades, and inducing the corresponding T cell response.

Acute chemokine stromal-derived factor 1α (SDF-1α, also known as CXCL12) stimulation induces PANX1 channel opening, cell polarization, and migration of CD4^+^ immature T cells (29, 48), as shown in **Figure 1**. Particularly SDF-1a recognition leads to a well-controlled PANX1 channel opening by a G protein-coupled receptor mechanism, leading to a rapid burst of ATP release, and subsequent focal adhesion kinase (FAK) phosphorylation (29). In this context, SDF-1α promotes translocation of P2X_4_ and mitochondria to the leading edge, increasing Ca^2+^ influx and pseudopod protrusion needed for cell polarization and migration (48). Polarized at the back of the cells, P2Y_11_ receptors are also activated, triggering inhibitory signaling *via* cAMP/PKA activation (**Figure 1**), and preventing mitochondria activity at the cell rear (66). Thus, polarized migratory T cells might present mitochondria at both leading edge (48), and/or at the cell rear (67), suggesting that specific chemical (i.e. chemokine treatments) and physical (i.e. adhesion of the surface) cues of the microenvironment differentially shape intracellular organelle location and migratory function. In fact, supporting the role of PANX-mediated signaling in T cell polarity, protein-protein interaction of PANX1 and collapsin response mediator protein 2 (CRMP2), indirectly controls microtubule stability (31). Interestingly, CRMP2 polarization towards the cell rear is required for T cell chemotaxis (68).

In freshly isolated naïve T cells subsets, CD4^+^ and CD8^+^ cells, PANX1 is abundantly present at the plasma membrane, whereas PANX2 abundance is much lower (65). Therefore, it is conceivable that upon stimulation the abundance of PANX2, and/or PANX3, could increase under specific conditions. Accordingly, *in vivo* data in murine model of experimental autoimmune encephalomyelitis disease suggests that stimulated T cells require functional PANX channels to transmigrate into the spinal cord (29, 50). PANX contribution during cell migration of specific T cell subsets remains largely unknown, but there is evidence that regulatory T cells required PANX1 to downregulate the response of effector T cells *in vivo* in a model of allergic airway inflammation (47). The latter data is very provocative, because it suggests that when PANX1 acts as a cell communication effector protein, it contributes to limit immune cell recruitment (47). Alternatively, PANX1 could promote T cell infiltration when is acting as a migration effector protein, likely by sustaining local signaling, which subsequently permits the polarization of the actin cytoskeleton or microtubule stability. Whether this migratory *versus* cell communication role of PANX1 is affected by the expression of other PANXs, or whether this is a mechanism present in other cell types is yet to be explored.

### 2.3 Dendritic Cells

Immature dendritic cells (DCs) normally reside in peripheral tissues where they scan the microenvironment in search of danger signals (69, 70). After antigen uptake, DCs migrate to secondary lymphoid organs, to initiate adaptive immune responses (69, 70). Resident DCs constantly internalize extracellular material, by phagocytosis or micropinocytosis, but upon danger signal detection these processes are downregulated and the migratory strategy changes from slow/random to fast/persistent (6, 30). Purinergic signaling is key for DCs response, as several P2X and P2Y receptors are expressed. In particular, P2X_7_ receptor together with PANX1, are required to sustain fast migratory phases (30, 32, 71). Transient exposure to high extracellular ATP concentrations activates P2X_7_ receptor, opening PANX1 channels to release ATP establishing an autocrine loop (32). This autocrine loop triggers Ca^2+^ influx *via* P2X_7_, calmodulin kinase II (CaMKII) activation and F-actin cytoskeleton to the cell rear (**Figure 1**), and is necessary to sustain DC fast migration (32). Interestingly, CaMKII directly controls the opening of PANX1 channels providing a direct link between purinergic and Ca^2+^ signaling that might be responsible for maintaining DCs migration (72). PANX1 appears to be equally distributed in migrating DCs, suggesting that this protein does not need to polarized to control DC speed. Since CaMKII regulates actin dynamics (73), its activity directly impacts on acto-myosin contractility, which is required for DC migration (30, 74). Thus, PANX1 contributes to DC migration by sustaining Ca^2+^ signaling *via* P2X_7_, which activates CaMKII maintaining actin polarization at the cell rear and subsequent contractility.

Activating signals trigger a maturation program in which several phenotypic changes, including the decrease of macropinocytosis, are required for the fast migration of DCs (6, 69). Interestingly, in other cells (i.e. neuroblastoma) ATP induces the internalization of PANX1 into macropinosomes (75), but it is not known whether the exact mechanism takes place during DC maturation and migration. Upon maturation, DCs increase the expression of cell surface molecules related to antigen presentation and directional migration, such as CCR7 chemokine receptor (6, 69). Directional migration in DCs largely depends on CCL19/CCL21 activation of CCR7 with the concomitant activation of CX43 channels (34). In the same line, migratory DCs increase the expression of CX43 and CX45 during homing to lymph nodes (35). Conversely, PANX1 channels were dispensable for this response as shown by the lack of effect observed with PANX blockers (34).

These data suggest that PANX1 channels might contribute to the early stages of DC migration upon danger signal detection, and that later stages including CCR7-dependent chemotaxis relies in the activity of CXs. This putative functional distinction between CXs and PANXs during DC migration might rely in different protein turnover at the plasma membrane, and/or the non-channel signaling function, as is shown for CX43 (76).

### 2.4 Monocytes and Macrophages

Although monocytes and macrophage originate from a common myeloid precursor and share several markers, they differ in their location, as monocytes are generally found in the bloodstream, while macrophages are tissue-resident cells. However, circulating monocytes differentiate into monocyte-derived macrophages (MMs) or monocyte-derived DCs (Mo-DCs) upon sensing of danger signals at the injury site (77). During CCL2-induced chemotaxis, monocytes release ATP, thus generating an autocrine loop with subsequent activation of P2Y6 receptors that is required for efficient migration (41), similar to other cells (see section *Dendritic Cells*, DCs). However, the molecular mechanism for ATP release was not elucidated. Monocytes, and macrophages, express both CXs and PANXs, but only CXs role has been shown during extravasation and migration. Particularly, CXs contribute by forming gap junction channels between monocytes and the endothelium (2, 40, 78). However, the functional role of PANXs expressed in monocytes (22), remains unaddressed.

Resident macrophages are named according to the different tissues in which they reside, although in general these cells share their primary functions: cellular detritus clearance, phagocytosis of pathogen particles, and in a lesser extent, antigen presentation (79). Resident brain macrophages, named microglia, quickly reacting to danger signals to prevent neuronal damage (80). ATP released during tissue damage acts as a chemoattractant of microglia *in vivo*, which extend their processes towards the injury site, a response that might require PANX1 channel activation (37). However, this need to be confirmed as only general blockers were used. Similarly, retinal microglia process extension induced by α-amino-3-hydroxy-5-methyl-4-isoxazole propionic acid (AMPA) receptor activation is prevented with Probenecid, a general PANX channel blocker (38). In addition, trovafloxacin another proposed PANX1 blocker (81), reduces microglia recruitment after traumatic brain injury (39). In a similar model PANX1 is required to induced the recruitment of microglia and other myeloid cells, and the lack of PANX1 improved the posttraumatic recovery of the mice (82). In addition, C5a-induced transmigration of microglia depends on PANX1-dependent ATP release, likely *via* an autocrine loop (39), such as the one shown in DCs (see section *Dendritic Cells*, DCs). However, this response might be specific different macrophage subsets, as PANX1 channels have only a mild contribution to C5a-induced chemotaxis in peritoneal macrophages, which was dependent on P2Y_2_ and P2Y_12_ receptors (36). Conversely, CX43 contributes to LPS-induced migration of peritoneal macrophages (83), suggesting that different channels might be required under other conditions.

Depending on the chemical cues of the microenvironment (i.e. cytokines), macrophages polarize to M1 or M2 macrophages, which exhibited different migratory properties (84). Interestingly, macrophage M1 polarization reduces PANX1 expression, whereas M2 polarization induces its upregulation (85). However, whether PANX channels play a role during the migration of these cells is still unknown.

## 3 Role of Tissue PANXs on Leukocyte Recruitment

Immune cell migration is not only induced by activation of PANX expressed in the migrating cell, but also can be indirectly promoted by PANX channels activated in the tissue. Initial observations by Chekeni et al, revealed that PANX1 channels were required for the release of “find me” signals (i.e., ATP and UTP) during T cell apoptosis, which triggered monocyte recruitment (86).

In liver, recruitment of monocytes is a hallmark of hepatic inflammation, involving apoptosis of hepatocytes induced by saturated free fatty acids (lipoapoptosis). Exposure to lipoapoptotic supernants elicits monocyte recruitment in an ATP-dependent chemokine-independent manner (87). PANX1 channels release ATP during lipoaptosis leading to c-Jun NH2-terminal kinase (JNK) activation in liver cells, revealing that hepatocytic PANX1 is key regulator of immune recruitment during nonalcoholic steatohepatitis (NASH) progression. In the same line, during obesity progression there is also accumulation of unsaturated fatty acids, which induce skeletal muscle inflammation and recruitment of immune cells. Thus, *in vitro* experiments with a muscle cell line that form myotubes show that treatment with palmitate induces ATP release from myotubes, which triggers monocyte recruitment (88). Interestingly, the release of ATP from the myotubes was independent of PANX1, but dependent on PANX3 channels (88). Consequently, myotubes that lack of PANX3 are unable to release ATP upon palmitate treatment, and do not trigger monocyte migration (88). A similar mechanism could occur during wound healing in a dorsal skin mouse model (89). PANX3 KO mice presented a delayed healing and inflammation resolution at the injury site. Indeed, the number of CD4^+^ T cells, neutrophils and macrophages was reduced in PANX3 KO mice, suggesting that tissue PANX3 was required for immune cell recruitment (89).

In the central nervous system the choroid plexus, located in the brain ventricle, is a key immune barrier between the cerebrospinal fluid and the blood. Epiplexus cells, resident innate immune cells of the choroid plexus, share markers and function with macrophages, DCs and other phagocytic cells (90). Under resting conditions epiplexus cells are sessile, but upon detection of extracellular ATP these cells increase their motility (91). This response depends on the ATP release *via* PANX1 channels from the epithelium of the choroid plexus, although epiplexus do not express PANX1 (91). Whether if during chronic inflammation or infection PANX expression is induced remains unknown.

Moreover, adipocyte-derived ATP release during adrenergic stimulation triggers macrophages recruitment (92). In addition, PANX1 opening is required for insulin-stimulated glucose uptake in adipocytes (93). Since insulin activates PANX1 channels causing the release of ATP, which in turn results in a signaling cascade indirectly allowing the transport of glucose into adipocytes, PANX1 might play a role in sustaining the inflammation observed during insulin resistance.

## 4 Role of PANXs on Migration of Non-Immune Cells

Unlike most immune cells that alter and deform little/transiently the extracellular matrix, mesenchymal cells require proteolytic enzymes to modify the microenvironment to undergo migration (5). Moreover, mesenchymal migratory cells use their actin cytoskeleton, form focal adhesion, and align with the extracellular matrix, with which and form focal adhesion (5, 10). However, despite these differences with amoeboid cell migration, mesenchymal cell migration also depends on acto-myosin contractility to move, although the motility of these cells is significantly slower (10). Another key protein for migration is the extracellular-signal-regulated protein kinase (ERK), a mitogen-activated protein kinase (MAPK), is a serine/threonine kinase, which modulates migration through phosphorylation of myosin light chain kinase (MLCK), calpain protease, paxillin, and focal adhesion kinase (FAK) (94). ERK activation can be triggered by cell matrix proteins (fibronectin, vitronectin, and collagen), growth factors (VEGF, FGF, EGF, insulin) and also indirectly by mechanical stress (94, 95). Indeed, mechanical stretch activates ERK through EGF receptor activation, triggering cell contraction (95). PANX1 channel opening is also affected by mechanical stress, although some evidence suggests that this occurs in an indirect fashion (96). However, regardless of the pathways activated PANX channel activity is affected by mechanotransduction and therefore these channels might contribute during cell migration and deformation of extracellular matrix, as it occurs during mesenchymal migration.

During the past years, increasing interest have grown on study PANX1 role during mesenchymal migration, particularly in the context of cancer progression (**Table 2**). For this reason, we decided to include in the following section, the latest publications associating PANX1 with non-immune cell migration.

**Table 2:** Summary of PANX1 contribution to non-immune cell migration.

Cell type	PANX1 effect on migration	Channel blockers//receptor inhibitors	P2R	Migratory stimuli	Migration techniques	Ref.
Astrocyte DITNC1 cell line	Increase	BBG, Apyrase	n.e.	Thy-1	2D wound healing	(97)
Cortical astrocytes (m)	Increase	n.e.	n.e.	Thy-1	2D wound healing	(97)
Dermal fibroblasts	No effect	PANX1 KO	n.e.	Wound	*In vivo* wound healing	(98)
I-10 Leydig tumor cell line	Increase	Cbx, Pbc, PANX1 siRNA, U0126	n.e.	None	2D wound healing, transmigration in transwells	(99)
MDA-LM2 and CN-LM1A breast cancer cells (h)	Increase	^10^PANX1, Cbx, Panx1 siRNA	n.e.	None	Counting of *in vivo* metastatic foci	(100)
BICR-M1Rk breast cancer cells (rt)	Increase	Cytochalasin B	n.e.	None	2D random migration	(25)
hTCEpi corneal epithelial cells (h)	Increase	^10^PANX1, BBG, NF157, Suramin, Apyrase, PPADS	P2X, P2Y	Electric field	2D galvanotaxis	(101)
N2a cells, neuroblastoma (m)	Increase	PANX1 siRNA	n.e.	Wound	2D wound healing	(26)
Rh18 eRMS, Rh30 aRMS cell line	Overexp. decrease migration	PANX1 loss of function mutants, AHNAK siRNA	n.e.	Wound	2D wound healing, 3D spheroid growth, *in vivo* tumor growth	(102, 103)
C6 glioma cells (rt)	Overexp. decrease migration	n.e.	n.e.	None	Transmigration in transwells, 3D spheroid growth	(104)
Keratinocytes from neonatal skin (m)	decrease	PANX1 KO	n.e.	Wound	*In vivo* wound healing	(98)
HDF (h), MDF (m)	decrease	Pbc, ^10^PANX1, PANX1 siRNA, A-740003,	P2X_7_	Wound	2D wound healing	(105)
	PANX3 effect on migration					
HDF (h)	decrease	PANX3 siRNA	P2X_7_	Wound	2D wound healing	(105)
	Increase		n.e.	Wound	*In vivo* wound healing	(89)
HaCaT keratinocyte (h)	Increase	PANX3 siRNA	n.e.	TFG-ß1	Transmigration in transwells	(89)

aRMS, Alveolar rhabdomyosarcoma; BBG, Brilliant blue G; eRMS, embryonal rhabdomyosarcoma; h, human; HDFs, human dermal fibroblasts; m, mouse; MDFs, murine dermal fibroblasts; Overexp, Overexpression; rt, rat.

### 4.1 Fibroblasts and Keratinocytes, Role of PANXs During Skin Cell Migration

The skin forms an active barrier and provides the first layer of defense by preventing the entry of foreign agents. This organ is a complex multilayer organization of cells with different but complementary functions, such as keratinocytes, melanocytes, fibroblasts, and immune cells (106, 107). During tissue damage skin resident cells communicate by using contact-dependent and independent mechanisms to quickly heal the wound (108). Consequently, ATP release and associated purinergic signaling play a key role during skin inflammation and wound healing (108). In particular, PANX1 is expressed in keratinocytes and fibroblasts from human and mice skin, as discussed below (98, 105, 109–111).

PANX1 levels decrease during adulthood, but increase after tissue damage (98). PANX1 is required for skin wound healing, as shown in a murine model of skin punch biopsies with a lack of PANX1 reduced tissue repair, but simultaneously increased fibrosis (98). Surprisingly, *in vitro* experiments revealed that the lack of PANX1 in keratinocytes increases their migratory potential. In contrast isolated skin fibroblasts from PANX1 knockout mice were more proliferative but showed decreased contractile properties in comparison to control conditions (98), suggesting that PANX1 expression is cell type specific, and that tissue interaction controls the overall migratory response in a cell-type specific and tissue specific manner. In the same line, PANX1 negatively regulates human dermal fibroblast migration when kept in monocultures. Indeed, fibroblasts lacking PANX1 or those treated with PANX1 channel blockers increase their speed of collective migration in wound healing assays, a similar but smaller response occurs when cells lack PANX3 (105). The decrease in cell migration during wound healing in fibroblasts is linked to decreased ATP release and activation of purinergic receptors. Consequently, P2X_7_ receptors blockade increases the speed of migration in human dermal fibroblasts (105). However, this last data should be considered carefully depending on the working model to be compared with, as the authors also report no effect over migration with P2X_7_ blockade in murine dermal fibroblasts (105). Additionally, *in vivo* experiments in a mice model reveal that PANX3 was required for proper wound healing (89). This suggests that different models of study might require different purinergic receptors. In any case, PANX1- and PANX3-dependent ATP release was consistently associated with a decrease in the collective and single migration of dermal fibroblasts, a response that relies in reorganization of the actin cytoskeleton (105).

These data reveal that PANX1 channels contribute to cell migration as a positive or negative regulators depending on the cell type and components of the microenvironment, including the available adenine nucleotides (**Figure 3**). However, the analysis of specific downstream signaling, and how the chemical cues of the microenvironment affect the role of PANX1 during migration remains largely unexplored.

### 4.2 PANXs Role During Astrocytic Migration Under Inflammation

Astrocytes are the more numerous glial cell in the brain, where these cells protect and feed the neurons (112). Astrocytes are crucial for tissue repairing during brain injury, and avoid spreading of neuronal damage by glial scar formation (112). During inflammation reactive astrocytes exhibit functional and morphological changes, as well as an increase in the expression of DAMPs receptors (112). Neuronal interaction with astrocytes controls cell migration *via* direct interaction of membrane proteins, such asThy-1 (CD90) (113, 114). Thy-1 is a membrane glycophosphatidylinositol (GPI) anchored protein that binds to αVβ3-containing integrin and syndecan-4 to stimulate FAK and actin reorganization (i.e. stress fibers formation), leading to morphological changes and migration in DITNC1 cell line. This astrocyte like cell line express high levels of αVβ3 Integrin and Syndecan-4, which resemble those observed in reactive astrocytes after tissue damage (115, 116). In DITNC1 cells Thy-1 stimulation triggers activation of intracellular signaling (PI3K and PLCγ) leading to Ca^2+^ release from intracellular stores opening CX43 and PANX1 channels. The ATP released *via* these channels activates P2X_7_ receptors and subsequent Ca^2+^ influx (117, 118), revealing that Thy-1 induction of DITNC1 cell migration depends on PANX1 channels.

**Figure 2:**
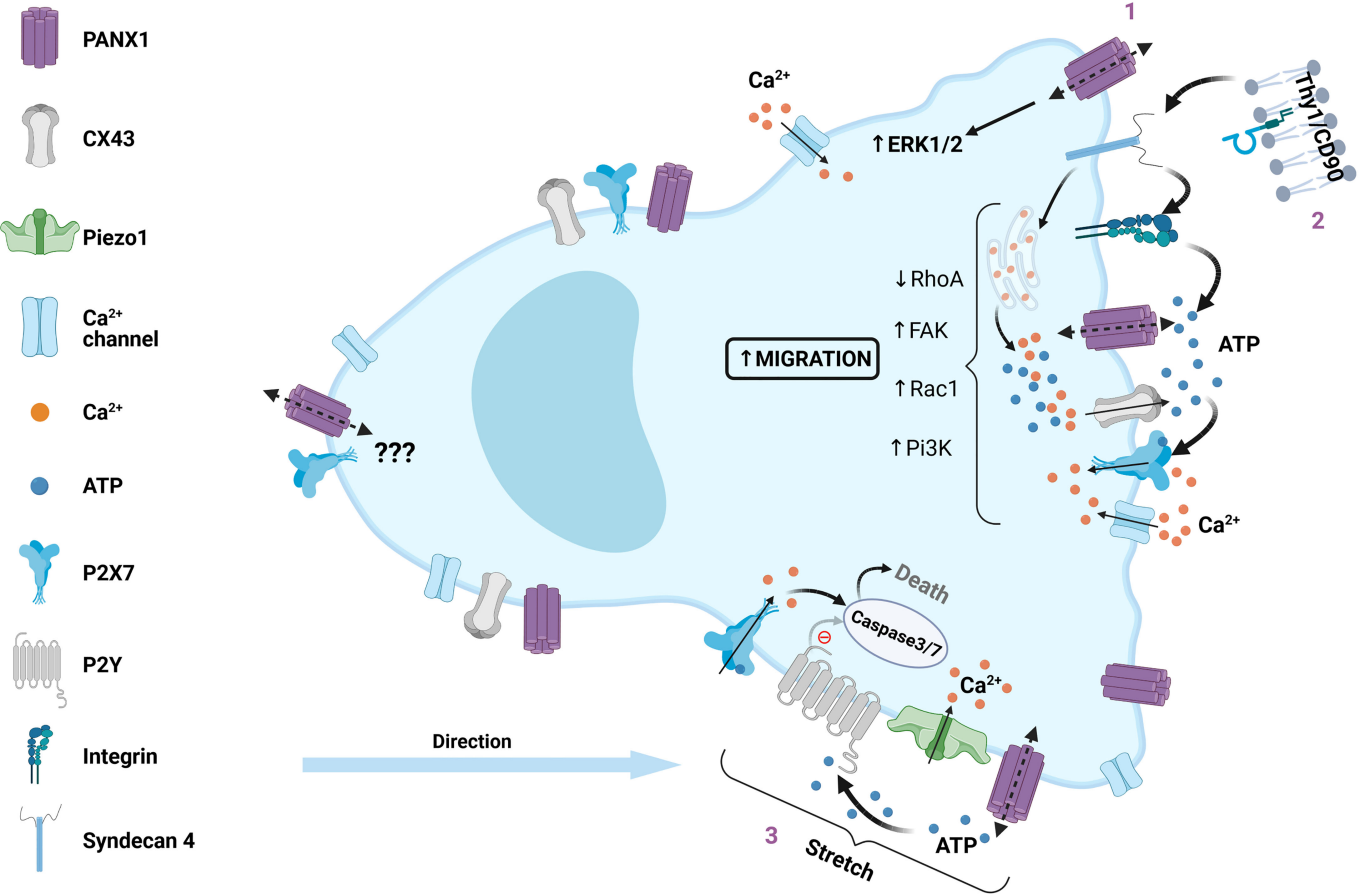
Role of PANX1 channels in mesenchymal cell migration. Intracellular regulation of PANX1 channels during migration of mesenchymal non-immune cells is described as a positive or negative regulation, here we summarize the positive regulation. (1) Increased cell migration is correlated with PANX1 overexpression *via* activation of ERK1/2 pathway, which could also play a major role during collective cell migration (not included in this figure). (2) Thy1/CD90, a surface protein expressed in normal cells, but overexpressed in certain pathologies, interacts with αVβ3-containing integrin and Syndecan 4, which promotes cell migration. The downstream signaling involves focal adhesion kinase (FAK) activation, PI3K, PLCγ and IP3 production, leading to Ca^2+^ release from intracellular stores, and opening of both CX43 and PANX1 channels. The latter promotes extracellular ATP release and subsequent P2X_7_ activation, Ca^2+^ influx and PKCα activation leading to Rac1 activation and RhoA inhibition. (3) Mechanical deformation during transepithelial migration lead to PANX1 channel activation, releasing ATP and activation of P2X_7_ receptors, leading to cell death by caspase 3/7 activation. Alternatively, PANX1 mutations that change its function lead to P2Y activation, preventing caspase activation and cell death.

Under resting conditions primary astrocytes express very low levels of αVβ3 Integrin and Syndecan-4, but their expression is induced during neuroinflammation (97). TNF is a cytokine associated with neuroinflammation and accordingly triggers expression of αVβ3 Integrin and Syndecan-4 in astrocytes, accompanied by PANX1, CX43, P2X_7_ receptors allowing the establishment of molecular toolkit required for Thy-1 signaling (97). Indeed, TNF-stimulated astrocytes respond to Thy-1, which leads to astrocyte cell migration by triggering PANX1 and CX43 ATP release and subsequent P2X_7_ receptor activation (**Figure 2**) (97), supporting the data obtained in DITNC1 cells.

In another pro-inflammatory context, reactive astrocytes derived from Amyotrophic Lateral Sclerosis (ALS) model hSOD1G93A transgenic mice present an increased abundance of several migration-related molecules, including αvβ3 Integrin, syndecan-4 proteoglycan, P2X_7_ receptor, PANX1, and CX43 (97). Thy-1 recognition induced both adhesion and migration of hSOD1G93A astrocytes (97). Intriguingly, TNF stimulation, and in ALS models triggers Thy-1 associated signaling expression, which pre-set a migratory phenotype in astrocytes, to which it is possible to speculate that, in general, pro-inflammatory conditions will induce a similar response preparing reactive astrocytes to migrate if needed, but whether PANX channels play a role during astrocyte migration in all pro-inflammatory conditions will require further studies.

### 4.3 Cancer Cells, Differential Contribution of PANXs to Tumor Progression

**Figure 3:**
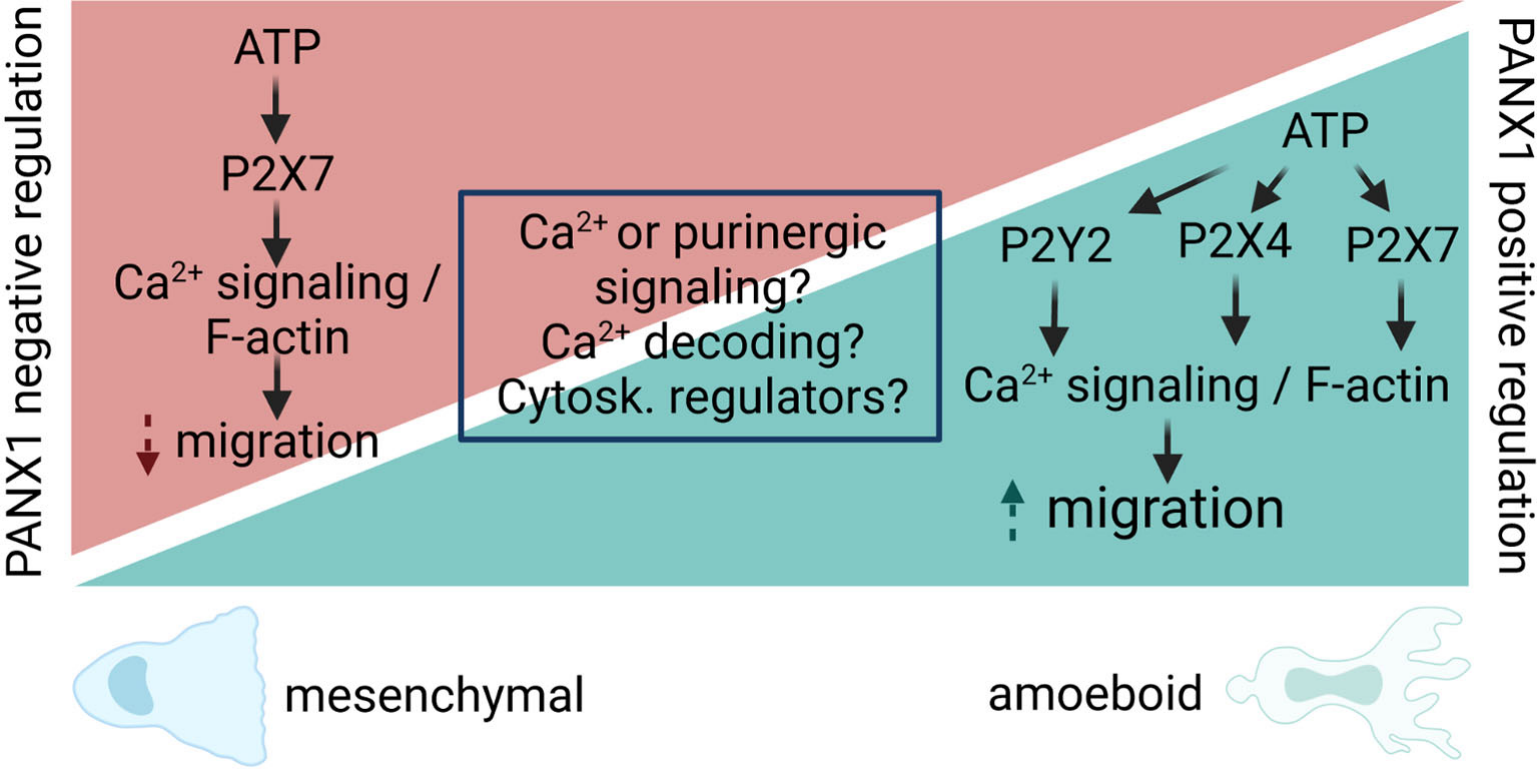
Proposed model for PANX1 channel regulation of cell migration. PANX1 channels act as positive or negative regulators of cell migration, despite the fact that some pathways and proteins are shared by those opposite responses. We propose that different outcomes are likely to occur due difference in Ca^2+^ and/or purinergic signaling (i.e. agonists, concentrations, etc). In particular, for Ca^2+^ signaling, the local (microdomain, nanodomain) regulation of it might activate different signaling cascades leading to increase or decrease of cell migration. In addition, the decoding of Ca^2+^ signals by Ca^2+^-sensitive enzymes (i.e. CaMKII) could directly modify the opening of PANX1, leading to changes in cytoskeleton dynamics directly or indirectly (i.e. cytoskeleton -Cytosk.- regulators) will result in signaling cascades that promote or inhibit cell migration. Therefore, the main contribution of PANX1 channels would be to amplify the initial response, and this would be downstream of the intrinsic cell-type specific Ca^2+^ response that ultimately determines the migratory outcome.

Tumor progression involves a series of sequential steps, which lead to tumor growth and metastasis. Cancer cell migration is the key step that allows invasion and colonization of new tissues. The stimulation of this response occurs during the epithelial to mesenchymal transition (EMT) (119, 120). Interestingly, dichotomic contribution of PANX1 on cancer cell migration has been reported (summarized in **Table 2**). Particularly, PANX1 can acts as a negative regulator for C6 cells motility, which are derived from rat glia. Indeed, overexpression of PANX1 reduces C6 glioma cell migration in different levels of complexity models of study (i.e. 2D, 3D spheroids, and *in vivo*) (104). In the same line a similar response is observed in rhabdomyosarcoma cells, in which inducible expression of PANX1 prevents cell migration (102). However, in this model PANX1 seems to play a role independent of its channel function, because it requires PANX1 physical interaction with AHNAK, a large scaffold protein (102, 103). Thus, at least in this rhabdomyosarcoma model it seems that PANX1 contribution might be related either to cytoskeleton re-organization and signaling, as shown for CX43 C-terminus (76). Interestingly, PANX1 expression induces gene and protein level upregulation of CX43, which has a tumor suppressive role in rhabdomyosarcoma (121). Altogether, these data supports the notion that PANX1 is a negative regulator tumor suppressor factor in cancer cells. However, in other cancer cell lines, PANX1 acts as a pro-migration factor as we discuss below (**Figure 3**).

Pioneer studies that revealed PANX1 interaction with the actin cytoskeleton, suggested its pro-migratory phenotype in breast cancer (25). Indeed, PANX1 promotes motility, transmigration, and *in vivo* invasion in melanoma, breast and testicular cancer cells: B16-BL6, B16-F10; CN34, CN-LM1A, MDA-MB-231 MDA-MB-468, MDA-LM2, and I-10 cells, respectively (99, 100, 122, 123). In some cases, such as testicular cancer PANX1 activity was required for ERK1/2 activity, E-cadherin and metalloproteinase 9 (MMP-9) revealing that these channels contribute with different aspects of cell migration (99).

Like normal fibroblasts (see section above), MDA-MB-231 cells are sensitive to Thy-1 that increases cell migration by releasing ATP *via* PANX1 channels with subsequent activation of P2X_7_ receptors in a positive feedback loop (123). Consequently, a mutation that increases the permeability of PANX1 channels leading to an exacerbated ATP release, also promotes cell motility, but not trans-endothelial migration (100), suggesting that PANX1 contributes to specific steps during tumor progression.

Which are the signals that lead to a positive or negative modulation of PANX1 on migration? As reviewed in this section, we hypothesize that it might depend on the cell type (**Table 2**), but the exact molecular mechanisms and signaling pathways that determine the outcome of the response remain unknown (**Figure 3**). The involvement of the other membrane channels that share functional and structural similarities with PANXs, such as the Leucine-rich repeat-containing 8 (LRRC8) proteins (124), is largely unexplored. Indeed, LRRC8A acts as a positive regulator of cancer cell migration (125), but how the activity of these channels affect the opening of PANXs, and how LRRC8 protein expression changes during cancer progression is not yet shown.

### 4.4 PANXs and Cell Migration During Aging, Senescence, and Neurodegeneration

Cellular changes progressively alter immune function during chronic inflammation and natural ageing, increasing susceptibility to infections and tumors (126). Associated with a chronic low-grade inflammatory state, is well accepted that cell motility decreases over time, which on immune cells might be due to accumulation of DNA damage after nuclear breakage (127). However, studies of cell migration in aging models are still scattered. Impaired phagocytosis and migration of DCs have been reported in aged humans (128). Not only cell decline (129), but also naive CD8^+^ and CD4^+^ T cell from old mice present lower migration and microtubules gene expression (130). Migration of aged marginal zone B cells at the spleen has also been reported to be impaired, consequently affecting immunoglobulin production (131). Human monocytes form elder volunteers showed altered gene profile of cellular motility (132), while bone marrow mesenchymal stem cells from aged human donors also present lower proliferation and migration abilities (133).

In the central nervous system, with smaller branches and slower motility process, microglia from aged mice exhibit reduced protrusion activity and cell migration after acute injury (134). During chronic neuroinflammation, aged mice of an Alzheimer’s disease model present increased mast cell infiltration in the brain parenchyma (51). Conversely, aged neutrophils migrate faster to the injury (135, 136), despite having impaired phagocytic and degranulation activity (137, 138). Similarly, myoblast have augmented migratory features (speed and directionality) during wound healing (139). Aging is likely to exert a cell-specific effect, and therefore it is hard to anticipate whether PANX1, and other PANXs, will contribute as a positive or a negative regulator as this will depend on the spatiotemporal regulation and accumulation of different ligands in the microenvironment.

## 5 Concluding Remarks

In the present review, we summarize the contribution of PANX channels during cell migration, emphasizing PANX1, that has been more widely studied. We have focused on immune cells as the integration of cell communication and cell migration is key for their function, despite mainly migrating as single cells. An interesting aspect of PANX-dependent purinergic signaling is the possibility that single migrating cells would have an impact on their neighbors. Using a mathematical model, Agliari et al. explored the hypothesis that ATP release and autocrine signaling during immune cell migration might impact neighboring cells while migrating as a group of single cells (140). The statistical inference approach revealed that migrating DCs have no instantaneous cell communication *via* release of small soluble molecules, such as ATP (140). However, the model only predicts immediate interactions and the release of adenine nucleotides or other small molecules will act with a delay considering the diffusion time and other parameters. The latter shows the need for the simultaneous study of cell communication and cell migration in a coordinated manner by using computational methods and theoretical frameworks, as it has been done for chemotaxis, or collective cell migration (141–143).

Another key aspect to link PANX1 and cell migration is the direct association between PANX channels and cytoskeletal components (actin, MyoII, microtubules), which are master regulators of cell migration. There is evidence of direct and indirect modulation of PANX channels by the cytoskeleton, which modifies membrane dynamics (144). In addition, PANX1 channels are somehow mechanosensitive, although in a lesser extent in comparison to other channels such as Piezo1 and Piezo2 (145). Still PANX1 could quickly react to changes in the membrane tension (96, 100, 146), providing a fast feedback mechanism in which the opening of the channel can be controlled by the mechanical cues of the microenvironment that surrounds the migrating cell. This could be sustained in time by the activation of enzymes that directly modify the opening of the channel, such as Ca^2+^-sensitive CaMKII (72), or by a positive feedback with P2 receptors (147, 148) (**Figures 2**, **3**). In addition, some evidence suggests a non-channel contribution of PANX1 during migration of rhabdomyosarcoma (102, 103), which has been observed in other channels that can act as signaling proteins such as CX43 (76), or as enzymes and therefore receive the name of ‘chanzymes’, such as TRPM7 (149).

PANX channels interplay with purinergic signaling and indirectly with Ca^2+^ signaling is well established (2, 30), but its direct contribution with Ca^2+^ influx seems to be cell specific (32, 51, 65, 150). Then, local Ca^2+^ signaling regulation could lead to different migratory outcome (**Figure 3**). Moreover, whether other ions, such as K^+^, could be relevant for the migratory is not yet demonstrated, although PANX1 channels directly contribute to migration induced by changes in the electric field, process named galvanotaxis (101). On the other hand, whether the opening of PANX channels contribute to ion flux and membrane voltage of the migrating cell is yet unexplored.

It is tempting to speculate about the necessity of PANX polarization during migration of leukocytes, but this should be carefully verified for each cell and stimuli. In neutrophils there is PANX1 polarization during migration (43, 86), but the same is not clear in T cells (29), and polarization seems to be not required for fast DC migration (32) (**Figure 2**). Therefore, the use of recently developed techniques, such as super resolution, optogenetics, and the development of new tools for live cell imaging monitoring of PANXs, will greatly improve our understanding of their role during cell migration.

Lastly, most of the studies have focused on PANX1, which seems to be a leading player during cell migration, but it is unclear the role of the other PANXs. Do PANXs have redundant functions? Are these cell-type specific? For example glioma cell migration is unaffected when changing the expression of PANX2, although cell growth was directly impacted (151). These data suggests that PANX2 and PANX3 might have other roles unrelated to cell migration and might be associated to cell growth and volume as recently reviewed (152). In the case of PANX3, which seems to act as a negative regulator of collective cell migration, it will be interesting to explore whether also prevent single cell migration. Finally, the role of PANXs has not yet been elucidated during chronic inflammation (i.e. obesity) or during aging (23, 152), conditions that change the responsiveness of immune cells (153). We propose that both conditions, chronic inflammation and aging, might induce the expression of different PANX isoforms in immune cells, leading to an increased migratory potential. However, how different PANXs isoforms orchestrate single and collective cell migration is still an unexplored field.

## Author Contributions

PH wrote large parts of the manuscript and provided advice for the figures. TL-L wrote some sections of the manuscript, and drafted the figures. AP contributed to drafting the manuscript. PS conceived the review, wrote and edited the manuscript, and revised the text and the figures. All authors contributed to the article and approved the submitted version.

## Funding

This project has received funding from the European Union’s Horizon 2020 research and innovation programme under the Marie Skłodowska-Curie grant agreement No 953489, ITN EndoConnect (PS), MINECO Spain PID2020-116086RB-I00 (PS), Forschungszentrum Medizintechnik Hamburg (FMTHH) 04fmthh2021 (PS), ANID Postdoctoral Project N° 3200342 (PH), MILENIO ICM-ANID P09-022-F (AP and PH).

## Conflict of Interest

The authors declare that the research was conducted in the absence of any commercial or financial relationships that could be construed as a potential conflict of interest.

## Publisher’s Note

All claims expressed in this article are solely those of the authors and do not necessarily represent those of their affiliated organizations, or those of the publisher, the editors and the reviewers. Any product that may be evaluated in this article, or claim that may be made by its manufacturer, is not guaranteed or endorsed by the publisher.
